# Beyond Cartilage Grafting in Rhinoplasty: Biopharmaceutical and Biomaterial Strategies for Nasal Cartilage Regeneration

**DOI:** 10.3390/life16101713

**Published:** 2026-10-13

**Authors:** Kyu-Ho Yi, Han Earl Lee, Hongseok Kim, Wonseok Choi, Jin Hyun Kim

**Affiliations:** 1 You and I Clinic, Seoul 06123, Republic of Korea; kyuho90@daum.net; 2 Opening Plastic Surgery Clinic, Seoul 06038, Republic of Korea; docearl@naver.com; 3 VOS Dermatology Clinic, 1–4F, 806 Eonju-ro, Gangnam-gu, Seoul 06022, Republic of Korea; drhong3@gmail.com; 4 V Plastic Surgery, Daegu 41940, Republic of Korea; plastsurgeon@naver.com

**Keywords:** nasal cartilage regeneration, rhinoplasty, biopharmaceuticals, polydeoxyribonucleotide, polynucleotides, extracellular vesicles, injectable hydrogel, controlled release, tissue engineering, 3D bioprinting, aesthetic medicine

## Abstract

Autologous septal, auricular, and costal cartilage remain the reference materials for structural and aesthetic rhinoplasty, yet each source is constrained by finite availability, donor-site morbidity, variable geometry, and the risks of warping, resorption, or revision. Nasal cartilage regeneration therefore represents more than a conventional tissue-engineering problem: a clinically useful product must recreate region-specific extracellular matrix, survive in a diffusion-limited environment, tolerate suturing and cyclic deformation, preserve the nasal airway, and maintain patient-specific contour under a thin soft-tissue envelope. This narrative review evaluates next-generation biopharmaceutical and biomaterial strategies for meeting those requirements. Direct evidence from human nasal chondrocytes and engineered nasal constructs is integrated with mechanistic evidence from broader cartilage regeneration, with explicit separation of nasal, auricular, and articular data. Chondrogenic proteins and small molecules, including transforming growth factor-beta, bone morphogenetic proteins, fibroblast growth factor 18, and kartogenin, can initiate matrix-forming programs but require spatial and temporal control to avoid fibrocartilage, hypertrophy, or mineralization. Polydeoxyribonucleotide and polynucleotide formulations have plausible anti-inflammatory and matrix-supportive actions, but evidence remains predominantly in vitro or articular; no direct clinical proof currently supports injection-only regeneration of structural nasal cartilage. Extracellular vesicles provide a cell-free signaling platform, whereas expanded nasal chondrocytes presently have the strongest tissue-specific translational lineage. Injectable collagen, fibrin, alginate, hyaluronic-acid, chitosan, gelatin-methacryloyl, and decellularized-matrix systems can localize these cargos, although biologic permissiveness often conflicts with immediate mechanical strength. Three-dimensional bioprinting and reinforced hybrid constructs offer patient-specific geometry, but clinical translation requires validated potency assays, suture retention, flexural testing, orthotopic safety, Good Manufacturing Practice-compatible production, and long-term objective assessment of shape and airflow. The most credible near-term applications are biologic enhancement of existing grafts, repair of small contained defects, and patient-specific partial-framework replacement rather than minimally invasive reshaping by regenerative injection alone. A stage-gated translational framework is proposed to align molecular potency, material performance, surgical usability, aesthetic precision, and durable nasal function.

## 1. Introduction

Cartilage is both a structural material and a biologically active tissue in rhinoplasty. The septal L-strut stabilizes the dorsal and caudal framework, the upper lateral cartilages participate in the internal nasal valve, and the lower lateral cartilages shape the tip, alar rim, and external valve. Aesthetic operations may appear to involve millimeter-scale changes, but those changes must remain stable against scar contraction, gravity, facial animation, respiratory cycling, and the intrinsic recoil of cartilage. For this reason, an apparently modest dorsal onlay, spreader graft, columellar strut, batten graft, or alar rim graft can have consequences for both contour and airflow. Autologous septal cartilage is generally preferred because it is biocompatible, readily carved, and obtained within the operative field; however, available volume is limited, particularly after previous septoplasty or rhinoplasty. Auricular cartilage offers useful curvature but has restricted dimensions, while costal cartilage provides volume at the cost of a second operative site and a recognized tendency toward warping and donor-site morbidity [1,2,3,4,5,6,7,8,9,10].

The limitations of conventional grafting have encouraged three overlapping regenerative strategies. The first is to manufacture replacement cartilage ex vivo from autologous cells. The second is to recruit host cells into a scaffold and induce cartilage formation in situ. The third is to use a biomaterial as a local drug-delivery depot that supports an existing graft, a small defect, or a patient-specific framework. These approaches are often grouped under the label of cartilage tissue engineering, yet they solve different clinical problems and face different regulatory burdens. A cell-expanded graft that replaces an absent alar segment is not equivalent to a nucleotide-containing hydrogel injected around diced cartilage, and neither is equivalent to a growth-factor-releasing dorsal scaffold. A clinically useful review must therefore connect molecular mechanism, delivery kinetics, material mechanics, manufacturing, and the exact rhinoplasty indication rather than treating regeneration as a single technology.

Human evidence demonstrates that nasal chondrocytes can generate clinically implantable tissue. A first-in-human observational study used autologous engineered nasal cartilage to reconstruct alar defects following tumor resection, establishing proof of principle that laboratory-grown nasal cartilage can survive and contribute to acceptable form and function [11]. Cultured autologous chondrocytes have also been applied in craniofacial or nasal augmentation and repair, and preliminary micrograft approaches have been described [12,13]. Nasal chondrocytes have additionally been used to repair knee cartilage, supporting their proliferative and chondrogenic potential outside the nasal environment [14]. Nevertheless, the human literature remains small, heterogeneous, and weighted toward reconstructive rather than elective aesthetic indications. Most proposed cargos—including fibroblast growth factor 18 (FGF18), kartogenin, polydeoxyribonucleotide (PDRN), polynucleotides (PN), and extracellular vesicles (EVs)—derive from articular-cartilage or in vitro models and cannot be assumed to produce a mechanically competent nasal graft.

This review examines biopharmaceutical and biomaterial strategies for nasal cartilage regeneration with an emphasis on aesthetic and reconstructive rhinoplasty. The central premise is that biologic activity alone is insufficient. A successful product must meet a target product profile that includes region-specific mechanical performance, reproducible geometry, local retention of therapeutic signals, absence of hypertrophy or calcification, surgical handling, airway compatibility, and durable contour. Direct nasal evidence is prioritized, while broader cartilage studies are used to explain mechanisms or identify transferable platform technologies. The review also defines realistic near-term use cases and proposes stage-gated criteria for clinical translation.

## 2. Literature Identification and Scope

A focused narrative search of PubMed/MEDLINE and publisher databases was conducted through 31 August 2026. Search combinations included nasal cartilage, septal cartilage, alar cartilage, rhinoplasty, tissue engineering, regenerative medicine, chondrocyte, mesenchymal stromal cell, extracellular vesicle, exosome, PDRN, polynucleotide, FGF18, kartogenin, controlled release, injectable hydrogel, bioink, and three-dimensional bioprinting. Direct studies of human nasal tissue, human nasal chondrocytes, orthotopic nasal models, engineered nasal implants, and clinical nasal reconstruction were prioritized. Mechanistic and platform evidence from articular and auricular cartilage was included when it addressed a defined translational question, such as chondrogenic signaling, hypertrophy, hydrogel design, printability, or extracellular-vesicle potency.

This is a narrative rather than systematic review. No prospective protocol, formal risk-of-bias instrument, or meta-analysis was used. To reduce overinterpretation, evidence is described according to its anatomical relevance: direct nasal clinical evidence, direct nasal preclinical evidence, craniofacial cartilage evidence, and indirect articular-cartilage evidence. The distinction is particularly important for drug-like interventions. Improvement in inflammatory markers or glycosaminoglycan synthesis in an osteoarthritis model does not establish the capacity to create a stable, suturable, load-bearing nasal framework.

## 3. Nasal Cartilage as a Distinct Regenerative Target

The nose contains several cartilaginous regions that differ in architecture and surgical function. Human septal cartilage is more hyaline than upper and lower lateral cartilage, with a higher relative abundance of type II collagen and lower abundance of types I and III collagen. The septum also demonstrates relatively high aggregate and tensile properties, whereas upper and lower lateral cartilage show distinct composition, crosslinking, and directional mechanical behavior [15]. Earlier compositional mapping demonstrated regional variation in collagen and sulfated glycosaminoglycan across the septum, supporting the surgical observation that the dorsal and caudal L-strut cannot be treated as an arbitrary piece of homogeneous cartilage [16]. Consequently, a construct intended as a septal replacement should not be qualified solely by histologic staining; it should reproduce the flexural stiffness, strength, thickness, and dimensional stability required for the intended portion of the L-strut.

The mechanical environment is unusual. Unlike articular cartilage, nasal cartilage is not designed primarily to withstand repetitive compressive joint loading. It experiences bending, tension, shear, suture-induced stress concentration, wound contraction, and respiratory deformation. The alar cartilages must be compliant enough to preserve natural motion yet sufficiently resilient to resist external valve collapse. Dorsal augmentation materials must resist bending and migration while avoiding visible edges under a thin soft-tissue envelope. A construct that is too soft may collapse or spread; a construct that is too rigid may look unnatural, erode overlying tissue, impair airflow, or create a palpable transition. Patient-specific geometry also creates a demanding tolerance: minor asymmetric swelling, contraction, or warping may be clinically visible even when biochemical properties appear acceptable.

Nasal cartilage is avascular, and its cells depend on diffusion from the perichondrium and adjacent tissues. This feature protects mature cartilage from some systemic immune influences but limits nutrient and oxygen delivery in thick engineered constructs. Cell density, hydrogel mesh size, construct thickness, matrix deposition, and maturation time therefore interact. Increasing polymer concentration can improve immediate stiffness while reducing diffusion and restricting cell-mediated remodeling. Conversely, a permissive low-density hydrogel may support matrix deposition but lack the strength required for implantation. The appropriate answer is often a composite architecture in which a temporary or permanent framework bears early load while cells synthesize a biologic matrix.

The intended clinical role determines acceptable degradation. A coating placed around diced cartilage may be expected to degrade after promoting cohesion and integration. A hydrogel that fills a small contained alar defect may need to persist until newly formed matrix becomes mechanically competent. A full septal implant may require prolonged reinforcement because even a mature cell-derived matrix may not immediately match the dimensions and flexural behavior of native cartilage. The degradation profile should therefore be linked to a measurable transfer of load from carrier to neotissue, not selected only for convenience of injection.

The target tissue is also exposed to a microbially colonized surgical field and lies close to thin skin and mucosa. Infection, extrusion, mucosal breakdown, and skin compromise are clinically meaningful failure modes. A material that performs well in a subcutaneous dorsal-mouse pocket may fail in an orthotopic septal or alar environment where fixation, motion, mucosal contact, and airway pressure are present. Orthotopic validation is therefore more informative than generic subcutaneous biocompatibility for late-stage development. The tissue-specific qualification requirements are summarized in Table 1.

## 4. Molecular Architecture of Nasal Chondrogenesis

### 4.1. TGF-Beta, BMP, and SOX9-Centered Matrix Formation

SOX9 is a central transcriptional regulator of chondrocyte identity and activates genes including COL2A1 and ACAN. In mesenchymal progenitors, transforming growth factor-beta (TGF-beta) signaling promotes condensation and chondrogenic differentiation through SMAD2/3-dependent pathways, while bone morphogenetic proteins (BMPs) can reinforce chondrogenesis through SMAD1/5/8. Classical pellet-culture studies established that bone-marrow-derived mesenchymal progenitors can enter a chondrogenic program in defined conditions [17,18]. Combinations such as TGF-beta2 and BMP-7 can enhance matrix formation by adipose-derived stromal cells [19]. However, the same signaling family can drive hypertrophy, fibrosis, or ossification depending on dose, duration, cell state, and mechanical context. For nasal applications, the therapeutic objective is not maximal expression of a single marker but stable production of region-appropriate cartilage without transition toward endochondral bone.

TGF-beta3 has particular relevance to nasal regeneration because controlled release from alginate microspheres within a poly(lactic-co-glycolic acid) scaffold induced dose-dependent orthotopic cartilage-like formation in a rat augmentation-rhinoplasty model [20]. This study illustrates a general principle: a cytokine can become clinically useful only when its local concentration and duration are engineered. A soluble bolus may diffuse rapidly, expose unintended tissue, and create a steep early peak; sustained release can extend recruitment and differentiation but may also prolong unwanted signaling. Release testing must therefore be paired with biological potency assays and spatial histology.

### 4.2. FGF18 and Small-Molecule Chondrogenic Modulation

FGF18 is a trophic factor for mature chondrocytes and progenitors [21]. The recombinant FGF18 analogue sprifermin increased femorotibial cartilage thickness in a randomized osteoarthritis trial, demonstrating that pharmacologic stimulation of cartilage anabolism can produce a measurable structural effect in humans [22]. This evidence is important but anatomically indirect. Intra-articular thickening of residual articular cartilage is different from de novo fabrication of a nasal strut. The nasal opportunity may be greatest as an adjunct that supports expanded chondrocytes or preserves matrix within a scaffold rather than as a stand-alone injection.

Kartogenin was identified through high-throughput screening as a small molecule capable of promoting chondrogenic differentiation and cartilage repair [23]. Small molecules offer advantages in synthesis, stability, and potentially tunable release, but they also act on broad intracellular networks and may have off-target effects. For nasal constructs, small-molecule candidates should be evaluated for maintenance of a non-hypertrophic phenotype, compatibility with the selected polymer and crosslinking chemistry, and retention of activity after sterilization or storage.

### 4.3. Inflammation, Matrix Catabolism, and Regenerative Timing

Inflammation is not merely a complication of implantation; it shapes cell fate and matrix turnover. Nuclear factor-kappaB activation can increase catabolic mediators, including matrix metalloproteinases and aggrecanases, while pro-inflammatory cytokines impair anabolic matrix synthesis. A regenerative implant placed into a revision rhinoplasty pocket may encounter fibrosis, altered vascularity, residual foreign material, bacterial biofilm, or chronic inflammatory signaling. Anti-inflammatory activity may therefore improve the local environment, but excessive immunosuppression could impair host defense and integration. Therapeutic design should distinguish short peri-implantation immunomodulation from prolonged suppression.

### 4.4. Hypoxia and Mechanotransduction

Low oxygen is physiologic for cartilage and can support chondrogenic differentiation. Micropellet culture under reduced oxygen enhances chondrogenic differentiation of human bone-marrow stromal cells [24]. At the same time, uncontrolled hypoxia in a thick construct can cause central cell death and heterogeneous matrix formation. Oxygen tension should therefore be treated as a spatial design variable, influenced by construct thickness, cell density, polymer content, and the timing of implantation.

Cells also interpret substrate stiffness, geometry, and nanoscale organization. Matrix elasticity can direct stem-cell lineage specification, while hydrogel mechanics, adhesive ligand composition, and geometric confinement modify cell fate [25,26,27,28]. These principles explain why increasing hydrogel stiffness is not invariably beneficial. A stiff network may improve handling but suppress cell spreading, matrix remodeling, or nutrient transport; a soft network may promote chondrogenic morphology while failing mechanically. Mechanically reinforced but biologically permissive composite constructs attempt to decouple these requirements.

### 4.5. Hypertrophy and Calcification as Critical Safety Endpoints

Mesenchymal stromal cells can undergo hypertrophic differentiation characterized by RUNX2, COL10A1, alkaline phosphatase activity, vascular invasion, and mineralization. Premature hypertrophy during in vitro chondrogenesis has been correlated with calcification after implantation [29,30]. Co-culture with nasal chondrocytes can suppress hypertrophy and has been associated with reduced in vivo calcification [31]. Parathyroid hormone-related protein and related pathway control have also been explored to limit hypertrophy [32]. For a nasal implant, even small calcified foci may alter flexure, become palpable, or distort contour. Histologic absence of mineralization in a short subcutaneous study is insufficient; long-term orthotopic imaging, bending tests, and section-wide quantification are required.

Figure 1 integrates these biological and mechanical control points. The central translational concept is balance: anabolic signaling must exceed catabolism long enough to build matrix, yet remain below thresholds that produce hypertrophy, fibrosis, or ectopic mineralization.

## 5. Biopharmaceutical Strategies

### 5.1. Recombinant Proteins and Controlled Morphogen Delivery

Recombinant proteins are attractive because their mechanisms can be defined and potency can be measured, but their short half-lives and concentration-dependent effects make delivery crucial. TGF-beta family members, BMPs, FGF18, insulin-like growth factor pathways, and combinations used in chondrogenic media can promote redifferentiation and matrix synthesis. In the 2026 hybrid nasal-septum study, human nasal chondrocytes encapsulated in fibrin around a medical-grade silicone framework were matured with BMP-2, insulin, and triiodothyronine, resulting in cartilage-marker expression and type II collagen deposition [33]. Such ex vivo exposure allows washing, release testing, and tissue qualification before implantation. In situ exposure is less controllable and should begin with lower-risk indications, limited volumes, and well-defined stopping rules.

A rational protein-delivery system should specify the active form, carrier binding, release mechanism, burst fraction, duration, residual activity, and local biodistribution. Adsorption to a polymer may denature a protein; photopolymerization may reduce activity; proteolysis may accelerate release; and newly synthesized matrix may trap or redistribute the cargo. Potency testing should therefore use released material rather than only the starting formulation. The endpoint should connect receptor activation to functional cartilage formation—for example, a validated cell assay followed by matrix production and mechanics—rather than relying solely on total protein content.

### 5.2. PDRN and PN: Plausible Adjuncts, Not Established Structural Regenerators

PDRN consists of deoxyribonucleotide polymers of defined source and size distribution, whereas PN products are related high-molecular-weight nucleotide formulations. Proposed mechanisms include adenosine A2A receptor activation after nucleotide degradation, support of nucleotide salvage pathways, modulation of inflammatory signaling, and promotion of repair-associated cellular activity. In collagen-induced arthritis, PDRN reduced cytokine production and disease severity through an A2A-receptor-associated mechanism [34]. In an in vitro osteoarthritis model, PN suppressed inflammatory responses and supported matrix synthesis [35]. A 2025 study incorporated PDRN into visible-light-crosslinked gelatin methacryloyl (GelMA), demonstrating sustained local delivery and enhanced cartilage-regeneration outcomes in a broader cartilage model [36]. Clinical observational work combining PN and hyaluronic acid in knee osteoarthritis has also been reported, but uncontrolled articular outcomes do not establish nasal structural regeneration [37].

These findings justify investigation, not clinical claims. There is no direct human evidence that PDRN or PN injection can regenerate a septal, alar, or dorsal cartilage framework. The regenerative burden in rhinoplasty is substantially higher than reduction in inflammatory biomarkers or modest matrix support. A structural graft must develop organized collagen, proteoglycan content, suture tolerance, flexural resilience, and shape stability. PDRN or PN may be more credible as an adjunct within a hydrogel that improves the early inflammatory environment, supports cell survival, or enhances integration of a conventional or engineered graft. The review therefore uses the term candidate biopharmaceutical rather than established chondrogenic drug.

Product-specific characterization is essential. Source material, purification, molecular-weight distribution, residual proteins or endotoxin, nuclease sensitivity, sterilization, and batch-to-batch consistency can alter biological activity. Because nucleotide formulations may be combined with cells, growth factors, or photocrosslinked polymers, interaction testing should assess whether they alter gelation, swelling, degradation, osmolarity, or cell viability. Dose-ranging should include a null or biphasic response, since more nucleotide is not necessarily more regenerative.

### 5.3. Extracellular Vesicles and Cell-Free Secretomes

EVs carry proteins, lipids, messenger RNA, and microRNA that can modify recipient-cell behavior. Mesenchymal-stromal-cell-derived exosomes have promoted osteochondral regeneration in preclinical models [38]. EVs enriched in miR-140-5p improved cartilage regeneration and attenuated osteoarthritis in rats [39], and stromal-cell-derived EVs promoted human cartilage regeneration in vitro [40]. In 2026, a three-dimensionally printed hydrogel containing miR-92a-3p-enriched platelet-rich-plasma-derived EVs was reported to enhance auricular cartilage regeneration [41]. These studies support a modular strategy in which EV cargo and a local hydrogel are optimized together.

The advantages of EVs are the absence of replicating donor cells and the possibility of off-the-shelf production. The disadvantages are biological heterogeneity, uncertain dose units, cargo drift with donor and culture conditions, rapid clearance without a carrier, and incomplete understanding of long-term effects. Particle number alone is an inadequate potency metric. A translational EV product should include identity markers, size distribution, purity, sterility, a mechanism-linked potency assay, and a release specification after incorporation into the final carrier. For nasal use, local persistence must be balanced against the possibility that prolonged signaling could create fibrosis, aberrant angiogenesis, or unintended chondrogenesis outside the target volume.

### 5.4. Nasal Chondrocytes, Mesenchymal Stromal Cells, and Co-Culture

Expanded autologous nasal chondrocytes have the strongest direct tissue-specific evidence. Human nasal septal chondrocytes can be expanded, redifferentiated in three-dimensional culture, and used to generate constructs with increasing type II collagen and native-like mechanical features after implantation [42]. Injectable alginate systems, porous constructs, and human-scale nasal-tip or auricular models have supported nasal chondrocyte viability and cartilage formation [43,44,45,46]. The first-in-human study by Fulco and colleagues used autologous nasal chondrocytes expanded from a biopsy and seeded onto collagen membranes to create grafts for alar reconstruction [11]. Although the cohort was small and the indication reconstructive, it remains a landmark demonstration that a nasal-cell product can traverse biopsy, expansion, tissue formation, surgery, and follow-up.

Mesenchymal stromal cells are easier to obtain in large numbers and can be incorporated into patient-specific constructs, as illustrated by adipose-derived cells in a cartilage-derived hydrogel and printed polycaprolactone framework [47]. Their disadvantages include donor variability, hypertrophy, and the need for stronger proof of stable cartilage identity. Co-culture combines the instructive phenotype of nasal chondrocytes with the expansion capacity or trophic support of stromal cells. Suppression of hypertrophy in nasal-chondrocyte/mesenchymal-cell co-culture is particularly relevant [31]. The optimal ratio, maturation period, and release criteria remain product-specific.

### 5.5. Combination Regimens

The most effective construct may combine a cell source, a short chondrogenic induction phase, an anti-catabolic adjunct, and a mechanically reinforced carrier. Combination increases the opportunity for synergy but also multiplies failure modes. A growth factor can change EV secretion; PDRN can alter inflammatory responses; polymer stiffness can change receptor signaling; and degradation can expose cells to a different mechanical environment. Development should therefore proceed through factorial experiments that identify the contribution of each component. A complex final formulation should not be adopted merely because each ingredient has an individually favorable literature (Table 2).

## 6. Injectable Hydrogels and Controlled-Release Systems

### 6.1. Natural and Semisynthetic Matrices

Hydrogels are central to cartilage regeneration because their hydrated networks resemble extracellular matrix and can entrap cells and drugs. Alginate gels under mild ionic conditions have supported three-dimensional culture of nasal chondrocytes, including injectable cartilage formulations [43,51]. Its limited intrinsic cell adhesion and relatively weak long-term mechanics often require modification or reinforcement. Fibrin is clinically familiar, adhesive, and cell-permissive; it can be molded around a framework and was used in the recent hybrid nasal-septum implant [33]. Fibrin hydrogels have also supported chondrogenic differentiation of human bone-marrow stromal cells, although formulation-dependent contraction and degradation remain important [52]. However, fibrin contracts and degrades rapidly unless composition, cell density, and reinforcement are carefully controlled.

Collagen provides native adhesion sites and is highly relevant to nasal-cartilage constructs. Human nasoseptal chondrocytes in collagen hydrogel have been bioprinted using freeform reversible embedding of suspended hydrogels (FRESH), producing homogeneous cartilage matrix after in vitro maturation [48]. Collagen’s low viscosity and slow gelation complicate unsupported printing, explaining the value of a temporary support bath. Hyaluronic acid and chondroitin sulfate provide cartilage-relevant biochemical cues and can be methacrylated or combined with gelatin to improve photocrosslinking and matrix mimicry [53,54,55,56]. Moldable thermosensitive chitin hydrogels provide another injectable route to three-dimensional cartilage formation [57]. Chitosan has favorable bioactivity and can form polyelectrolyte complexes with alginate; a 2026 nasal-chondrocyte study demonstrated sustained viability and increasing chondrogenic markers in alginate-chitosan composites [58].

GelMA is widely used because its cell-adhesive gelatin backbone can be photocrosslinked with tunable stiffness. A PDRN-loaded visible-light GelMA system illustrates its value as a drug depot [36]. Yet recent nasal-specific work showed a fundamental tradeoff: higher GelMA concentration improved stiffness and handling but reduced extracellular-matrix deposition and increased catabolic gene expression [50]. This finding argues against selecting the stiffest printable formulation. The desired early mechanical behavior may be better obtained from a printed framework or fiber reinforcement while the surrounding GelMA remains permissive to matrix formation.

Decellularized cartilage extracellular matrix can supply tissue-specific biochemical cues. Patient-specific nasal constructs have used cartilage-derived hydrogel within printed polycaprolactone frameworks [47]. Decellularized-matrix bioinks can promote lineage-relevant behavior, but their composition varies with donor tissue, decellularization, milling, and digestion [59,60]. Residual DNA, immunogenic proteins, endotoxin, and protease activity require specification. A material derived from articular or auricular cartilage may not fully reproduce nasal regional biology.

### 6.2. Crosslinking and Release Kinetics

Crosslinking chemistry determines injection feasibility, cell compatibility, swelling, degradation, and drug release. Ionic crosslinking is mild but can be reversible in physiological ion concentrations. Enzymatic systems may be biocompatible but difficult to standardize. Photocrosslinking enables spatial control and rapid fixation, although light dose, photoinitiator, and tissue exposure must be justified. Visible-light systems may reduce ultraviolet-related concerns, but the final formulation still requires cytotoxicity, residual initiator, and released-cargo testing.

Release may occur through diffusion, polymer degradation, affinity binding, or carrier-particle erosion. For small nucleotide fragments, rapid diffusion can dominate unless molecular entanglement or binding is sufficient. Proteins may bind to collagen, fibrin, or heparin-like domains, creating a release profile that changes as cells remodel the matrix. EVs can be physically trapped, electrostatically associated, or chemically linked; each method changes bioavailability. Release studies should be conducted in physiologically relevant media, at the intended construct thickness, and for the full period over which biological activity is claimed. Cumulative mass alone is insufficient; the biological activity of each fraction should be measured.

### 6.3. Mechanical Reinforcement and Shape Fidelity

Reinforcement strategies include thermoplastic lattices, printed microfibers, cages, meshes, and permanent or degradable frames. Covalent attachment of printed thermoplasts to gelatin hydrogels improves mechanical integration [61], while printed microfibers can reinforce otherwise soft hydrogels [62]. Polymer cages can reduce contraction of tissue-engineered cartilage [63]. These technologies are particularly relevant to the nose because a framework can preserve the intended contour during the months required for matrix maturation. Reinforcement must not create sharp transitions, pressure points, or stress shielding that prevents biological matrix from carrying load.

Suture pull-out, three-point bending, cyclic flexure, torsion, creep, and recovery after deformation are more informative for many nasal indications than compression alone. Tests should be performed at baseline, after hydration, during degradation, and after biological maturation. A construct may pass a single short-term bending test yet undergo slow creep or asymmetric contraction. Mechanical mapping across the construct is also important because patient-specific shapes contain thin edges, curves, and junctions that can be weaker than standardized coupons.

### 6.4. Injectable Systems: Realistic and Unrealistic Indications

An injectable system is most plausible when the defect is small, contained, and mechanically protected; when it is used as a coating or adhesive around an existing graft; or when it fills a reinforced mold. Injection alone cannot guarantee organized cartilage or a precise external contour. In an uncontained dorsal or tip pocket, a low-viscosity formulation may migrate, spread, or create nodularity. In situ crosslinking can improve localization but introduces risks of uneven light delivery, incomplete polymerization, thermal effects, and entrapment of air or blood. The term injectable should therefore describe delivery, not imply that major structural rhinoplasty can be replaced by a simple office procedure.

### 6.5. Critical Quality Attributes

A hydrogel-based nasal product should have predefined critical quality attributes: polymer identity and substitution degree; viscosity and injectability; gelation time; swelling; degradation; sterility and endotoxin; residual crosslinker or photoinitiator; drug loading and release; cell viability; matrix potency; dimensional accuracy; mechanical behavior; and packaging stability. These attributes should be linked to the clinical role. For example, suture retention is mandatory for a graft designed for open fixation but may not be required for a small contained injectable adjunct. Figure 2 summarizes the continuum from therapeutic cargo to carrier, structural format, and clinical role.

## 7. Three-Dimensional Bioprinting and Patient-Specific Constructs

Three-dimensional printing addresses one of the most difficult aesthetic requirements: reproducible patient-specific geometry [64]. A workflow can convert surface imaging or computed tomography into a digital target, subtract the baseline anatomy, design a desired implant, and fabricate a mold, framework, or cell-laden construct. A patient-specific engineered nasal implant combining a printed polycaprolactone architecture with a cartilage-derived hydrogel and human adipose-derived stromal cells maintained shape and generated cartilage-like tissue after subcutaneous implantation [47]. The study established a persuasive engineering workflow but did not demonstrate human rhinoplasty efficacy.

Bioprinting with human nasoseptal chondrocytes has advanced from proof of printability to in vivo stability. FRESH printing of chondrocyte-laden type I collagen produced customizable constructs with relatively homogeneous matrix [48]. Subsequent work examined maturation and in vivo stability [49]. Nasal-specific GelMA experiments showed that printability and strength must be balanced against cell-mediated matrix formation [50]. The 2026 fibrin-silicone construct extended the hybrid concept to a full-scale L-strut-like framework, with in vitro matrix deposition, subcutaneous stability, and cadaveric surgical handling [33]. Together, these studies shift the field from generic cartilage disks toward anatomically relevant constructs.

The main technical constraints are printing-induced cell stress, resolution, diffusion, maturation time, and interfacial weakness. Smaller nozzles improve resolution but increase shear stress. High polymer concentration improves print fidelity but reduces cell remodeling. Thick constructs can develop oxygen and nutrient gradients. Framework-hydrogel interfaces can delaminate unless geometry or chemistry produces mechanical interlocking. A mature matrix may also contract and distort the original print. Reinforced hydrogel studies, cages, multi-material printing, and long-term auricular models provide useful engineering solutions [53,61,62,63,65,66,67,68,69,70,71,72], but the nasal target is typically thinner, more mechanically heterogeneous, and more visually unforgiving.

Clinical manufacturing adds another layer. The digital workflow must control image acquisition, segmentation, design approval, printer calibration, material lot, cell density, maturation, and dimensional inspection. Aesthetic intent must be formally documented because the desired postoperative surface is not simply a copy of missing anatomy. For elective augmentation, the construct represents a designed change; for reconstruction, it may reproduce a contralateral or pre-defect contour. Surgeon review of the digital design is therefore a critical manufacturing step rather than an informal preference. Figure 3 provides a conceptual illustration of visual interpretation, observer-to-subject perspective transformation, and right-left labeling in clinical assessment.

Preimplantation release testing must avoid destroying the only patient-specific graft. Non-destructive imaging, culture-supernatant biomarkers, standardized witness coupons, and process validation may be combined. However, witness coupons are informative only if they experience the same cell density, crosslinking, nutrient conditions, and maturation as the implant. Dimensional inspection should include local curvature and edge thickness, not only global volume. Representative translational studies are summarized in Table 3.

## 8. Translation to Aesthetic and Reconstructive Rhinoplasty

### 8.1. Current Human Evidence

The direct clinical evidence is encouraging but limited. Fulco et al. reported an observational first-in-human trial in which autologous engineered nasal cartilage reconstructed alar defects after tumor resection [11]. The tissue was generated from a small nasal-cartilage biopsy, expanded, seeded on a collagen scaffold, and matured before implantation. The study demonstrated feasibility and acceptable one-year outcomes in a small cohort, but it did not compare the construct with standard autologous grafts and did not address elective dorsal augmentation. Yanaga et al. described clinical use of cultured autologous auricular chondrocytes for craniofacial or nasal augmentation and repair [13]. Gentile et al. reported preliminary alar reconstruction using autologous chondrocyte micrografts and platelet-rich plasma [12]. These experiences support continued development while underscoring the absence of large comparative trials.

### 8.2. A Hierarchy of Credible Clinical Roles

The near-term role with the lowest biological burden is enhancement of an existing graft. A hydrogel containing an anti-inflammatory or trophic cargo might improve cohesion of diced cartilage, reduce early catabolism, or support integration at the graft-host interface. Such an adjunct still requires evidence that it does not increase infection, fibrosis, irregularity, or resorption. The next level is repair of a small, contained defect where surrounding native structures protect the construct. Partial-framework replacement—such as a defined alar segment or limited septal component—requires stronger mechanics and fixation. Full L-strut or major dorsal replacement has the highest burden because failure can affect both appearance and airway.

Patient-specific augmentation occupies a special category. It may not replace missing tissue but must create a stable, intentionally altered contour. The product should be compared with accepted augmentation materials and autologous cartilage, not merely with an empty control. Aesthetic precision must be quantified using standardized photography and three-dimensional surface imaging. Functional assessment should include validated nasal-obstruction measures and, when relevant, objective airflow or valve measurements. A biologically active implant cannot be considered successful if it creates an attractive dorsal line while compromising breathing.

### 8.3. Target Product Profiles

Each indication should begin with a target product profile. A small alar insert may require curvature, flexibility, edge taper, and resistance to notching. A septal extension graft requires suture retention, bending stiffness, and resistance to long-term deformation. A dorsal implant requires surface smoothness, fixation, and very low visibility under soft tissue. A coating around autologous cartilage may prioritize injectability, adhesion, anti-catabolic action, and complete resorption. These products should not share a single generic mechanical specification.

### 8.4. Clinical Endpoints

Early feasibility studies should prioritize safety and technical success: infection, extrusion, skin or mucosal compromise, airway obstruction, reoperation, abnormal calcification, and progressive deformation. Objective three-dimensional geometry should be captured immediately after swelling has resolved and at serial long-term time points. A useful analysis separates global volume retention from local curvature and asymmetry. Standardized palpation or ultrasound may identify edge visibility, nodules, or calcified foci. Patient-reported satisfaction is important but is susceptible to expectation and cannot replace objective assessment.

For comparative studies, the comparator should match the indication. Autologous septal or auricular cartilage is appropriate for small structural grafts, costal cartilage for high-volume reconstruction, and a commonly used synthetic implant for selected augmentation settings. Outcomes should be assessed at least 12 months, with 24 months preferable for shape stability, resorption, calcification, and revision. Blinded photographic assessment should be complemented by surgeon-rated handling and intraoperative modification, because a product that cannot be safely trimmed, sutured, or repositioned is unlikely to be adopted.

### 8.5. A Stage-Gated Development Sequence

A six-stage development sequence is proposed: target product profile, in vitro qualification, orthotopic preclinical testing, Good Manufacturing Practice process validation, first-in-human feasibility, and comparative clinical evidence. Each stage includes a mandatory go/no-go gate. This framework prevents premature clinical translation based solely on favorable staining or short subcutaneous studies. A construct should not progress unless potency correlates with functional matrix, mechanics remain acceptable during degradation, and orthotopic testing excludes warping, extrusion, mineralization, and airway harm.

## 9. Safety, Manufacturing, and Regulatory Considerations

Biological safety depends on the component class. Autologous nasal chondrocytes have low alloimmune risk but can dedifferentiate during expansion, acquire senescence-associated changes, or vary between donors. Mesenchymal stromal cells add concerns about hypertrophy, ectopic tissue formation, and poorly defined identity. EVs are non-replicating but can carry variable signaling cargo and require rigorous purity and potency testing. Recombinant growth factors can diffuse beyond the construct or induce unwanted fibrosis, angiogenesis, or ossification. PDRN and PN products require source, purification, molecular-weight, endotoxin, and residual-protein controls. Combining components does not average their risks; it creates new interactions that must be tested in the final formulation.

Material safety includes degradation products, extractables, leachables, residual catalysts, crosslinkers, photoinitiators, and sterilization-related changes. A permanent reinforcing framework may reduce early deformation but can become a locus for infection, extrusion, or chronic mechanical irritation. A degradable framework must retain strength until neotissue assumes load and then degrade without acidic or inflammatory microenvironments. Cadaveric handling studies, such as those used for the full-scale hybrid septal construct, are valuable for fit and workflow but cannot predict long-term biological performance [33].

Manufacturing must control both biological and geometric variability. For autologous products, the process begins with biopsy quality and continues through transport, cell isolation, expansion, passage number, culture supplements, maturation, release, and return to the operating room. A patient-specific printed product adds image segmentation, design transfer, print parameters, post-processing, and dimensional verification. Critical process parameters should be linked to critical quality attributes. For example, nozzle diameter and pressure affect cell viability and resolution; polymer substitution and light dose affect stiffness and residual initiator; maturation time affects matrix content and possibly contraction.

Regulatory classification will depend on jurisdiction and product composition. A cell-expanded implant, a drug-eluting scaffold, an EV-loaded hydrogel, and a permanent hybrid framework may be regulated through different or combined pathways. Early dialogue with regulators is advisable because the primary mode of action, degree of manipulation, autologous versus allogeneic use, and presence of a permanent device influence requirements. The development program should be designed around the final product rather than postponing classification until after proof-of-concept experiments.

Elective aesthetic use requires particular attention to risk tolerance. A patient seeking augmentation may have normal airway function and no tissue deficit. The acceptable uncertainty is therefore lower than in oncologic reconstruction or severe trauma. First-in-human studies should begin with indications in which the potential benefit justifies surgical and biologic uncertainty, use independent safety oversight, and include predefined criteria for removal or revision. Marketing language should not imply cartilage regeneration when the evidence demonstrates only temporary volume, inflammation reduction, or matrix-marker expression.

Long-term surveillance is essential because warping, calcification, resorption, and chronic infection may emerge after the typical duration of an early feasibility study. Registries or structured post-market follow-up should document product lot, manufacturing deviations, imaging, functional outcomes, and revision findings. Explanted constructs provide rare but valuable information and should undergo histology, microbiology, mechanical examination, and component-specific analysis.

## 10. Research Priorities

Several experiments would substantially improve the field. First, nasal-region-specific target values should be expanded beyond bulk composition to include three-point bending, tensile anisotropy, suture pull-out, cyclic deformation, creep, recovery, and failure at thin edges. These data should be stratified by septal, upper lateral, and lower lateral region and by age. The design criteria reported for human nasal cartilage provide a strong foundation [15], but reproducible interlaboratory methods are needed.

Second, candidate biopharmaceuticals should be tested in human nasal chondrocytes and clinically relevant co-cultures before they are promoted for rhinoplasty. PDRN, PN, EVs, FGF18, kartogenin, and other cargos should undergo dose–response testing for COL2A1, ACAN, type II collagen, glycosaminoglycan, catabolic enzymes, COL10A1, alkaline phosphatase, and mineralization. Mechanistic assays should be connected to construct-level mechanics. A treatment that improves matrix gene expression but weakens the graft should not advance.

Third, delivery studies should compare soluble cargo, hydrogel incorporation, microparticle release, and framework-associated delivery using the same dose. Release fractions should be tested for biological activity. Spatial mapping should determine whether matrix forms uniformly or only near the construct surface. For EVs, potency should be defined by a mechanism-linked assay rather than particle count alone.

Fourth, orthotopic models should reproduce the intended operation. Dorsal augmentation, alar replacement, and septal reconstruction impose different loads and fixation. Studies should include a clinically relevant comparator, standardized surgical pockets, objective three-dimensional follow-up, airway assessment, and sufficient duration to detect deformation and mineralization. Subcutaneous models remain useful for screening but should not be treated as definitive translational evidence.

Fifth, clinical studies should separate reconstructive and elective aesthetic indications. Early trials should report the tissue source, cell passage, formulation, maturation, implant dimensions, fixation, antibiotic protocol, adverse-event definitions, imaging schedule, and revision threshold. Outcomes should combine safety, three-dimensional morphology, nasal function, patient-reported benefit, and surgeon usability. Publication of negative and explant data will be particularly important in preventing repetition of avoidable failures.

Finally, development should favor the simplest product that can meet the clinical need. A cell-free graft coating may reach practice sooner than a fully bioprinted septum, while a mature autologous chondrocyte sheet may be more predictable than a highly complex mixture of cells, EVs, growth factors, and nucleotides. Complexity is justified only when it improves a predefined performance gap.

## 11. Conclusions

Nasal cartilage regeneration has progressed from laboratory cartilage disks to autologous human reconstruction, controlled morphogen delivery, patient-specific printed frameworks, nasal-chondrocyte bioinks, and full-scale hybrid septal prototypes. The field now has sufficient mechanistic and engineering depth to move beyond generic claims of chondrogenesis. The decisive question is whether a product can maintain precise shape and nasal function while generating stable, non-hypertrophic cartilage in a surgically usable format.

Expanded nasal chondrocytes currently provide the strongest tissue-specific biological foundation. Growth factors, small molecules, PDRN/PN, and EVs are promising modulators, but most evidence remains indirect and should be framed as hypothesis-generating. Injectable hydrogels are best understood as localization and microenvironment technologies, not as proof that major structural rhinoplasty can be achieved by injection alone. Reinforced and bioprinted systems offer patient-specific geometry but require rigorous mechanical, orthotopic, manufacturing, and long-term clinical validation.

The most credible translational sequence begins with graft-supporting adjuncts and small contained defects, progresses to partial-framework replacement, and reserves full structural replacement or elective patient-specific augmentation for products that pass stage-gated safety and performance criteria. Alignment of molecular potency, release kinetics, matrix quality, surgical handling, aesthetic precision, and airway preservation will determine whether next-generation cartilage regeneration becomes a durable component of rhinoplasty rather than an attractive but clinically incomplete concept.

## Figures and Tables

**Figure 1:**
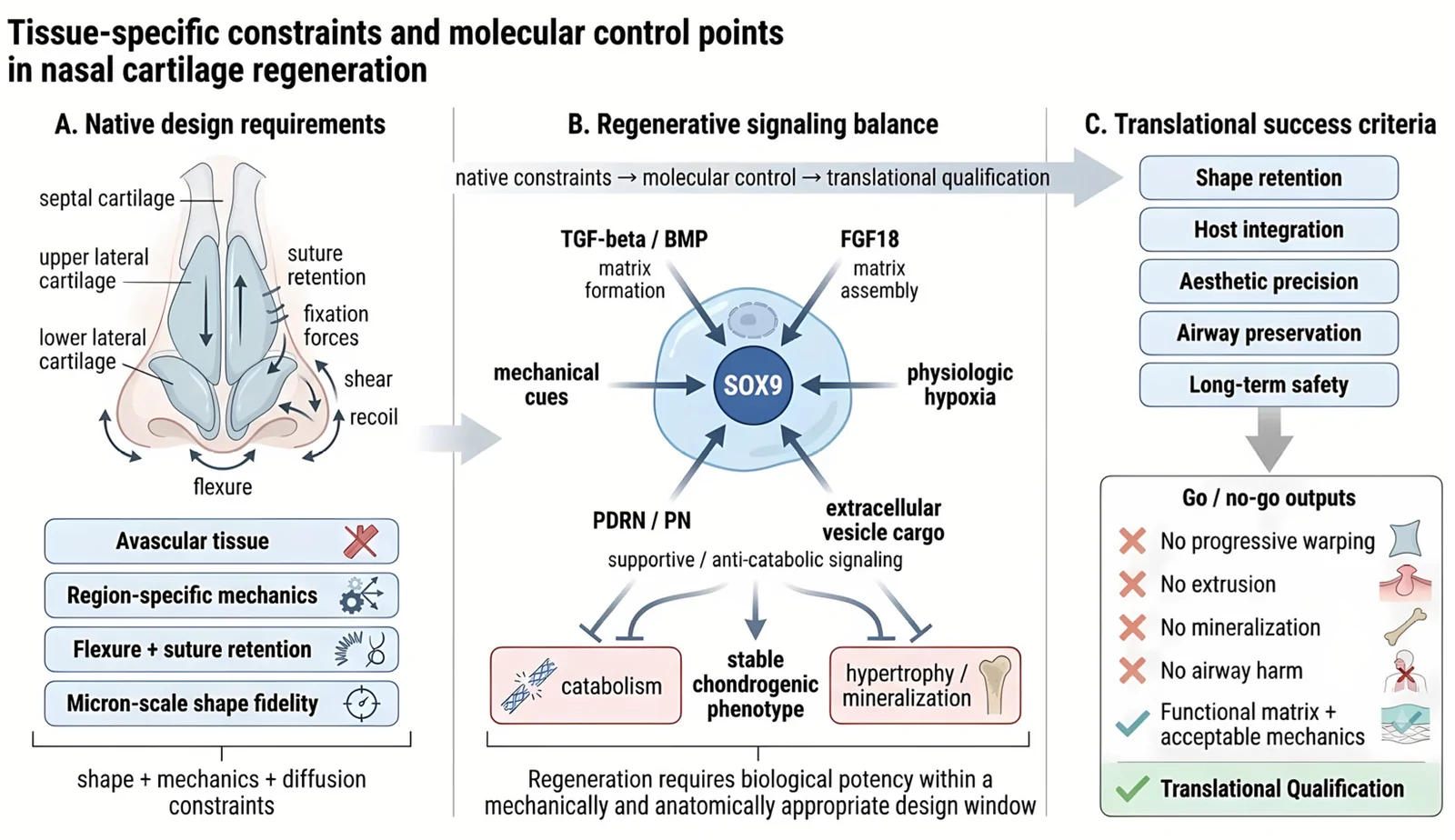
Tissue-specific constraints and molecular control points in nasal cartilage regeneration. Anabolic TGF-beta/BMP, FGF18, hypoxic, mechanical, nucleotide, and extracellular-vesicle signals converge on a stable SOX9-centered phenotype. Translation requires region-appropriate mechanics, surgical handling, host integration, aesthetic precision, airway preservation, and explicit exclusion of catabolism, hypertrophy, and mineralization. Abbreviations: ACAN, aggrecan; ADAMTS5, a disintegrin and metalloproteinase with thrombospondin motifs 5; BMP, bone morphogenetic protein; COL, collagen; EV, extracellular vesicle; FAK, focal adhesion kinase; FGF18, fibroblast growth factor 18; HIF-1alpha, hypoxia-inducible factor 1 alpha; MMP13, matrix metalloproteinase 13; PDRN, polydeoxyribonucleotide; PN, polynucleotide; TGF-beta, transforming growth factor beta; YAP/TAZ, Yes-associated protein/transcriptional coactivator with PDZ-binding motif. Created by the authors; executable script supplied. Created by the authors using Biorender.

**Figure 2:**
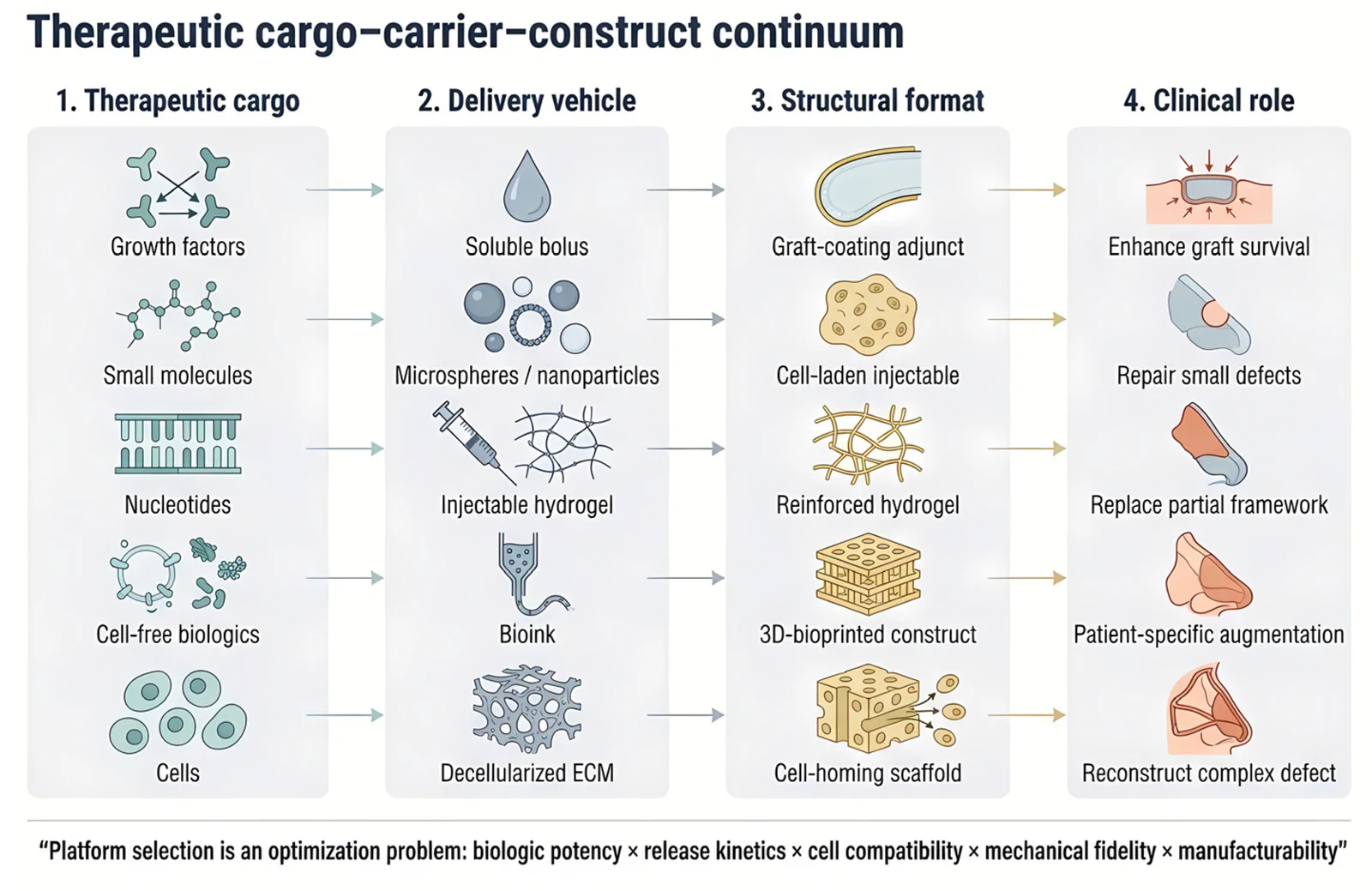
Therapeutic cargo–carrier–construct continuum. A regenerative product should be designed from the intended clinical role backward. The same cargo may require a different carrier, release profile, and structural format when used to enhance an autologous graft, repair a small defect, replace part of the nasal framework, or create patient-specific augmentation. Abbreviations: BMP, bone morphogenetic protein; ECM, extracellular matrix; EV, extracellular vesicle; FGF18, fibroblast growth factor 18; MSC, mesenchymal stromal cell; PDRN, polydeoxyribonucleotide; PN, polynucleotide; TGF-beta3, transforming growth factor beta 3. Created by the authors using Biorender.

**Figure 3:**
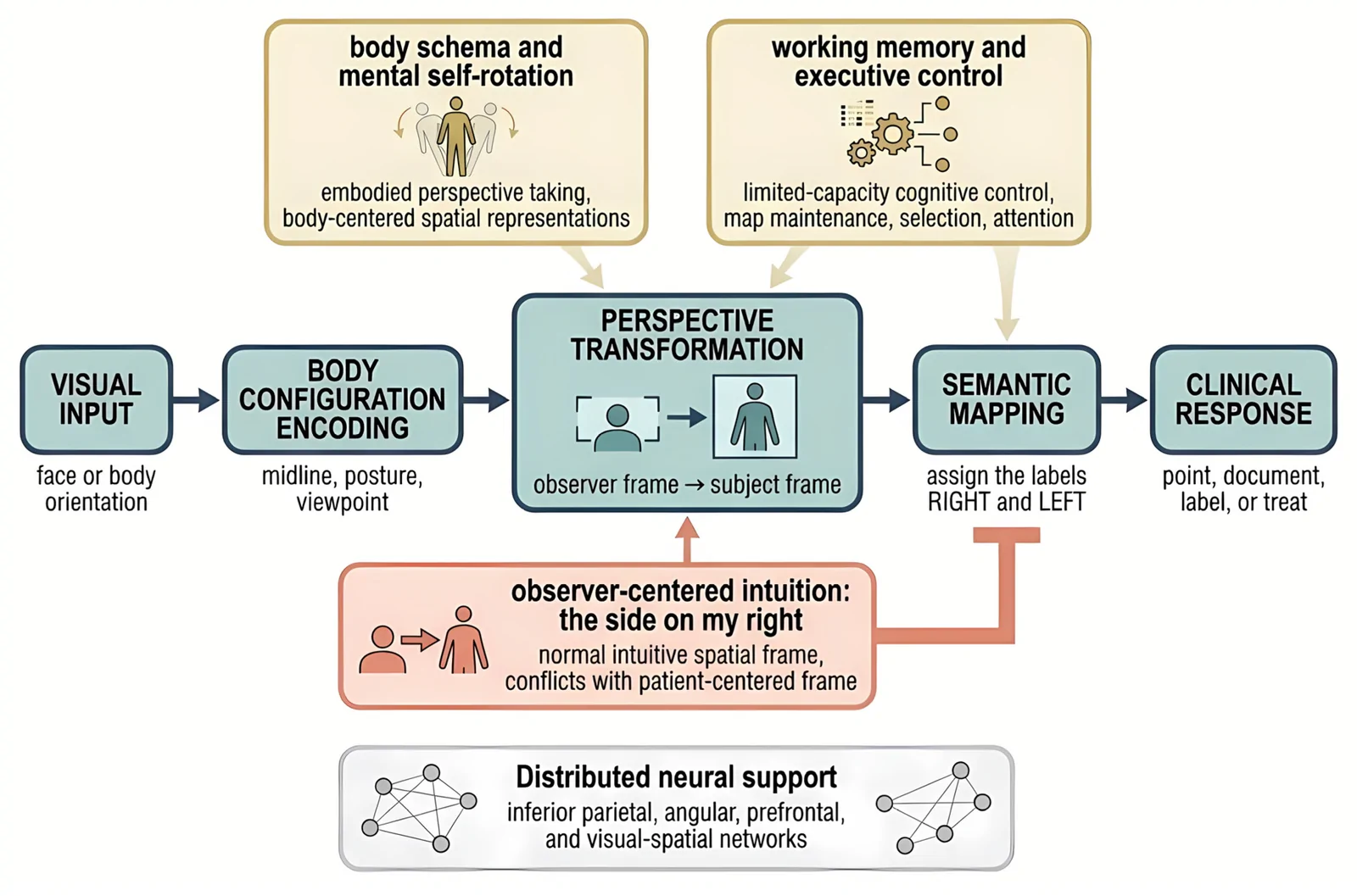
Conceptual schematic of spatial orientation and perspective transformation in clinical assessment. The diagram traces visual input, body-configuration encoding, observer-to-subject perspective transformation, semantic assignment of right and left, and clinical response. It depicts body schema, mental self-rotation, working memory, executive control, and distributed neural support, together with the potential conflict between observer-centered intuition and the patient-centered reference frame. This schematic concerns spatial interpretation rather than the six-stage product-development sequence described in Section 8.5. Created by the authors using Biorender.

**Table 1:** Tissue-specific target product requirements for regenerative nasal cartilage.

Requirement	Clinical Rationale	Suggested Qualification	Failure Signal
Region-specific geometry	Millimeter-scale asymmetry or edge prominence can be visible under thin nasal skin.	3D dimensional inspection; curvature and edge-thickness mapping	Contraction, local bulging, asymmetry, visible step-off
Flexural performance	Septal, dorsal, and alar grafts face bending, recoil, and scar contraction.	Three-point bending; cyclic flexure; creep and recovery	Progressive warping, collapse, permanent set
Suture and handling	Many structural grafts must tolerate fixation, trimming, and repositioning.	Suture pull-out; tear testing; simulated carving/fixation	Tearing, fragmentation, interface delamination
Biological identity	Matrix-marker expression alone may coexist with fibrocartilage or hypertrophy.	COL2A1/ACAN/type II collagen; matrix distribution; COL10A1/mineralization	Type I-dominant matrix, calcification, heterogeneous necrosis
Integration and surface safety	The construct lies close to mucosa and thin soft tissue.	Orthotopic histology; microbiology; skin/mucosal assessment	Extrusion, chronic inflammation, infection, erosion
Nasal function	Aesthetic contour must not compromise the internal or external nasal valve.	Airway examination; validated obstruction score; airflow testing when indicated	New obstruction, valve collapse, mucosal injury

**Table 2:** Biopharmaceutical classes and their translational position in nasal cartilage regeneration.

Strategy	Representative Evidence	Potential Nasal Role	Principal Limitation
TGF-beta/BMP delivery	Sustained TGF-beta3 release induced orthotopic cartilage-like formation in an augmentation-rhinoplasty model [20].	Cell recruitment; ex vivo maturation; local chondrogenic induction	Dose-dependent fibrosis, hypertrophy, or ossification
FGF18/sprifermin	Trophic effects in chondrocytes and increased articular-cartilage thickness in a randomized trial [21,22].	Matrix support for cells or grafts	Articular evidence does not prove de novo nasal framework formation
Kartogenin	Small-molecule chondrogenic activity and cartilage repair in preclinical systems [23].	Stable, controllable cargo for local release	Off-target signaling and uncertain nasal phenotype
PDRN/PN	Anti-inflammatory and matrix-supportive effects; PDRN-GelMA cartilage platform [34,35,36].	Adjunctive local immunomodulation or cell support	No direct structural nasal-regeneration trial
EVs/secretome	MSC- and miRNA-enriched EVs promote cartilage repair in preclinical studies [38,39,40,41].	Cell-free trophic signaling in hydrogel carriers	Heterogeneous cargo, potency, dose, and persistence
Nasal chondrocytes	Preclinical constructs and first-in-human alar reconstruction [11,33,42,48,49,50].	Autologous engineered grafts and bioinks	Biopsy, expansion time, cost, donor variability
MSCs/co-culture	Patient-specific constructs and suppression of hypertrophy in nasal co-culture [31,47].	Scalable cell source and trophic support	Hypertrophy, identity, and lot-specific potency

**Table 3:** Representative translational evidence for engineered nasal and craniofacial cartilage platforms.

Study/Platform	Model	Key Observation	Translational Interpretation
Fulco et al. [11]	First-in-human; autologous engineered nasal cartilage for alar reconstruction	Feasible implantation with acceptable form and function in a small cohort	Strongest direct clinical proof of concept; not comparative and not elective augmentation
Chang et al. [42]	Human nasal chondrocyte neocartilage implanted in mice	In vivo maturation increased type II collagen and produced native-like features	Supports preimplant maturation and tissue-specific cells
Mendelson et al. [20]	Orthotopic rat augmentation scaffold with sustained TGF-beta3	Dose-dependent cartilage-like tissue formation	Demonstrates controlled morphogen delivery and cell homing
Yi et al. [47]	Patient-specific PCL framework, cartilage-derived hydrogel, human adipose-derived cells	Shape maintenance and cartilage-like tissue after subcutaneous implantation	Integrates digital design, reinforcement, and biologic matrix
Lan et al. [48,49]	FRESH-bioprinted human nasoseptal chondrocytes in collagen	Custom shape, homogeneous matrix, and subsequent in vivo stability testing	Nasal-specific cell-laden biofabrication platform
Perry et al. [50]	GMP-grade GelMA with human nasal chondrocytes	Higher stiffness accompanied lower matrix deposition and more catabolic signaling	Mechanical strength and biological permissiveness must be decoupled
Perrier-Groult et al. [33]	Silicone-fibrin-human nasal chondrocyte hybrid septal implant	Matrix formation, subcutaneous stability, full-scale fabrication, and cadaveric fit	Advanced hybrid prototype; orthotopic and clinical validation remain required

## Data Availability

No new data were created or analyzed in this narrative review. The figure-generation code is supplied with the manuscript files.
